# Wide-Awake Local Anesthesia with No Tourniquet (WALANT) Carpal Tunnel Release in the Clinic: A Clinical Practice Update

**DOI:** 10.3390/jcm14186407

**Published:** 2025-09-11

**Authors:** T. Hunter Stocker-Downing, Rebecca McAllister, Sean Chan, Ian Mullikin, Kevin Krul

**Affiliations:** 1 Department of Orthopaedic Surgery, Tripler Army Medical Center, 1 Jarrett White Rd., Honolulu, HI 96819, USA; 2 Department of Orthopaedic Surgery, Queens Medical Center, Honolulu, HI 96813, USA; 3 Franciscan Orthopedic Associates at St. Anthony Hospital, Gig Harbor, WA 98332, USA; 4 Trinity Medical Center, Williamsville, NY 14221, USA

**Keywords:** WALANT, carpal tunnel, hand surgery, local anesthesia, clinic surgery

## Abstract

**Background:** Wide-awake local anesthesia with no tourniquet (WALANT) carpal tunnel release (CTR), performed in the clinic setting, has emerged as a safe, efficient, and cost-effective alternative to traditional operating room (OR)-based decompression. With increasing adoption in clinic settings, WALANT CTR offers the potential to improve access, reduce costs, and maintain excellent patient outcomes. **Purpose:** This clinical practice update provides an evidence-based summary of clinic-based WALANT CTR, including patient selection, procedural setup, safety profile, cost implications, and system-level considerations for implementation. **Recent Findings:** Multiple prospective and retrospective studies confirm the safety of WALANT CTR in the clinic setting, with complication rates comparable to OR-based procedures and no increase in surgical-site infections when field sterility is used. Cost analyses report a 70–85% reduction in facility costs per operative case, and patient satisfaction remains consistently high, even among those with anxiety disorders or psychiatric conditions. Adjunctive interventions such as virtual reality technology devices and noise-canceling headphones further enhance the awake surgical experience. Institutional adoption remains variable, with barriers including sterility concerns, billing uncertainty, and credentialing logistics. This clinical update offers detailed, practical guidance on implementing WALANT CTR for surgeons and staff, covering scheduling, staff training, clinical integration, billing, and compliance considerations. **Summary:** Clinic-based WALANT CTR is a high-value, patient-centered approach supported by a growing body of literature. With appropriate patient selection, streamlined workflows, and institutional support, this model can optimize surgical care delivery in both resource-rich and limited environments.

## 1. Introduction

Carpal tunnel syndrome (CTS) is the most common peripheral nerve entrapment, affecting an estimated 3–5% of adults. It imposes a significant healthcare burden, with over 500,000 surgical decompressions performed annually in the United States and total insurer costs exceeding USD 1 billion [1,2]. In North America, the majority of procedures are still performed in traditional OR settings under sedation or general anesthesia, contributing to high per case costs and limited access. As healthcare systems seek more cost-effective, patient-centered delivery models, interest in clinic-based, wide-awake approaches has grown.

The wide-awake local anesthesia with no tourniquet (WALANT) technique was popularized by Don Lalonde in Canada in the early 2000s [3]. Originally developed to minimize anesthetic risk in complex patients, WALANT rapidly gained traction due to its procedural efficiency, high patient satisfaction, and minimal resource requirements. In the U.S., adoption remained limited until the 2010s, when cost-containment pressures and increased evidence of safety drove broader acceptance. National data show an increase in WALANT CTR from 1.2% to 3.4% of cases between 2010 and 2020 [2]. More recently, the COVID-19 pandemic has accelerated interest in office-based surgery across many specialties, positioning clinic-based WALANT CTR as a scalable alternative to the traditional OR model.

In a 2020 American Society for Surgery of the Hand (ASSH) survey, 79% of respondents reported performing WALANT procedures, and 62% currently incorporate the technique into their practice. Approximately 24% of these are performed in non-OR clinic settings, with 43% of surgeons offering WALANT for all carpal tunnel releases [4]. In a busy practice, an open operating room can quickly be taken up with multiple CTR given the sheer abundance of pathology. With more practices implementing minor procedure rooms and more surgeons offering WALANT CTR, it seems like a logical evolution to move CTR to the procedure room instead of using valuable OR time. This is a big leap for many, as the transition to doing surgery leaves many questions unanswered. The lack of standardized guidance or institutional framework has left many hand surgeons hesitant to change their practice. However, there is a huge potential benefit for surgeons, patients, and the overall cost to healthcare.

With many hand surgeons considering clinic-based CTR, there are several factors to be considered when adapting to this change. Patient selection, clinic setup, billing, complications, outcomes, and cost will be covered in this clinical practice update. We will provide a comprehensive review of common concerns, followed by an example clinical implementation of clinic-based WALANT CTR.

## 2. Materials and Methods

A literature review was conducted to synthesize current evidence regarding wide-awake local anesthesia with no tourniquet (WALANT) carpal tunnel release (CTR) performed in the clinic setting. A formal search of the PubMed/MEDLINE database was performed through July 2025 using the following keyword combinations: “WALANT,” “carpal tunnel release,” “wide-awake surgery,” “clinic-based hand surgery,” “field sterility,” and “office-based CTR.” Boolean operators (AND, OR) were used to combine terms and identify relevant studies. The search was limited to English-language articles involving human subjects. The literature review was conducted independently by three reviewers, encompassing studies published from 2010 onwards, with articles screened for relevance based on predefined inclusion criteria.

Inclusion criteria were original research studies, systematic reviews, cost analyses, implementation studies, and surveys that specifically addressed clinic-based WALANT CTR or related wide-awake procedures performed outside the operating room. Studies focusing exclusively on WALANT performed in traditional OR settings were excluded unless they contributed relevant safety or procedural insights applicable to office-based environments. Reference lists of included articles were also reviewed to identify additional studies of relevance.

A total of 112 articles were initially identified. After screening titles and abstracts for relevance, 41 full-text articles were reviewed. Of these, 18 met inclusion criteria and were used to inform this clinical practice update. Included studies ranged from prospective trials and retrospective cohorts to survey-based implementation studies and cost-effectiveness analyses. Where multiple articles addressed similar outcomes (e.g., safety, patient satisfaction, or cost), the most comprehensive or recent studies were prioritized. Artificial intelligence tools, GenAI, were utilized to support aspects of writing and editing in preparation of the manuscript. All content was critically reviewed, verified and finalized by the authors to ensure accuracy, originality and adherence to academic standards.

In addition to the literature review, real-world clinical practice considerations were incorporated based on the authors’ collective institutional experience performing WALANT CTR in both military and civilian outpatient clinic environments. These experiential insights were used to supplement areas where evidence may be evolving, such as patient selection, anesthesia technique, and workflow optimization.

## 3. Patient Selection

Successful implementation of clinic-based WALANT CTR depends on proper patient selection. The literature supports the following criteria for inclusion (Table 1):

- ASA Physical Status I–II (minimal systemic disease);
- Idiopathic CTS confirmed by clinical exam and/or electrodiagnostics;
- No prior wrist/hand surgeries or anatomical complexities;
- No significant psychiatric conditions that would impair cooperation;
- Patient acceptance of an awake procedure and the ability to tolerate the clinic environment.

Of note, WALANT CTR has been utilized in patients who may not be cleared readily for traditional surgery due to underlying medical comorbidities and the risks of anesthetic administration. However, these wide-awake techniques are still typically used in the operating room due to increased availability of intraoperative monitoring. The selection criteria proposed above pertain to the clinic-based WALANT CTR.

Of note, anxiety disorders or psychiatric conditions are not absolute contraindications [5,6]. In fact, recent studies suggest that patients with generalized anxiety disorder or depression report similar pain and satisfaction outcomes between WALANT CTR and traditional operating room-based decompression.

## 4. Patient-Reported Experience and Outcomes

Patient satisfaction with WALANT CTR is consistently high, with greater than 90% of patients expressing a preference for WALANT if undergoing surgery again [7,8]. In a series of 1011 WALANT procedures, Rhee et al. found a 98% satisfaction rate with minimal complications [8]. A prospective comparison found no difference in intraoperative pain (VAS scores) between clinic-based and OR-based CTR. Additionally, clinic procedures were significantly shorter in duration in terms of total time in the facility (78.6 min vs. 215.7 min; *p* < 0.01) and met return-to-activity milestones quicker [9].

In a United Kingdom study using the Hospital Anxiety and Depression Scale (HADS), patients who underwent WALANT CTR reported no increase in anxiety pre- or post-procedure, and none required anxiolytic medication [10]. Patients with underlying psychiatric conditions, including anxiety or depression, did not experience worse outcomes compared to the general cohort [5,6].

## 5. Institutional Barriers and Implementation

Despite increasing support in the literature for WALANT CTR, implementation by surgeons varies widely. In a national survey of hand surgeons by Gunasagaran et al., 30–40% of respondents reported hesitancy in adopting WALANT regularly, citing concerns over infection control, sterility protocols, medico-legal liability, and billing complexity [11,12]. These challenges were particularly prominent in transitions to clinic-based procedures, where institutional policies and infrastructure may limit sterile field setup or anesthetic administration outside the operating room. Successful implementation occurred in practices in which administrative leadership, nursing staff, and surgeons were educated on WALANT protocols, and place-of-service billing and regulatory compliance were clarified in advance [12]. These findings suggest the critical importance of institutional buy-in and interdisciplinary training as essential components of sustainable adoption in clinic-based CTR workflows.

## 6. Global and Low-Resource Applications

WALANT techniques are increasingly adopted worldwide, particularly in limited-resource and outreach settings where access to anesthesia support and a formal operating room is limited. For example, a UK cohort demonstrated safe, reproducible CTR outcomes in a field-sterile clinic environment with no adverse events and high patient satisfaction [10]. Another study conducted in East Africa found that the implementation of WALANT techniques reduced surgical costs and increased access for patients in low-income settings. The training for local surgeons and staff was initiated with support from a non-governmental organization via mentorship and hands-on workshops led by experienced surgeons [12]. The minimal setup required for WALANT makes it ideal for mission settings, community hospitals, and clinics seeking to optimize procedural access without increasing infrastructure resources and associated expenses.

## 7. Technical Considerations in Clinic-Based WALANT CTR

Clinic-based carpal tunnel release under WALANT differs from standard operating room procedures in terms of setup, instrumentation, and anesthesia delivery. Despite these differences, several studies confirm that these adjustments do not compromise safety or effectiveness when appropriately implemented [3,9,11].

Implementation of clinic-based WALANT CTR requires adherence to field sterility principles, clinical workflow efficiency, and appropriate documentation for reimbursement. Field sterility has been validated as a safe and efficient alternative to full operating room sterility protocols. LeBlanc et al. conducted a multicenter prospective trial of carpal tunnel surgeries performed using sterile gloves, a single drape, and a minor surgical tray, with no increase in infection rates compared to traditional OR procedures [11]. Other required equipment includes lidocaine with epinephrine, chlorhexidine prep, and protective eyewear. This minimalist setup is widely accepted in WALANT practice and facilitates high procedural throughput in the clinic setting.

Anesthetic technique typically involves the use of buffered lidocaine with epinephrine, injected subcutaneously along the planned incision line and deep into the carpal tunnel. Although its use is not obligatory, epinephrine serves to induce local vasoconstriction and minimize intraoperative bleeding, thereby eliminating the need for a tourniquet. Additionally, its use prolongs the duration of local anesthetic, enhancing postoperative recovery. No complications have been reported regarding epinephrine use in WALANT CRR [13]. A delay of 20–30 min is recommended to achieve optimal hemostasis from epinephrine before incision [3]. No sedation or IV access is required [3]. Visual inspection of the surgical field prior to incision confirms adequate vasoconstriction. Although a tourniquet may be tolerated by some patients for short periods of time, avoidance enhances patient comfort.

Instrument trays are similarly streamlined. A minor hand tray consisting of a scalpel, small retractor, scissors, forceps, and nerve hook is sufficient for most cases [9]. Closure is often performed with absorbable subcuticular sutures or adhesive strips to minimize follow-up requirements.

Postoperatively, patients are provided with written instructions and typically discharged within 15–30 min. Most patients do not require formal hand therapy, and follow-up may be conducted virtually or by phone in uncomplicated cases. An example of a traditional workflow can be found below.

## 8. Complications and Safety

Clinic-based WALANT CTR has demonstrated equivalent safety to OR-based procedures. In cohorts totaling over 400 procedures, complication rates were under 3%, with zero surgical-site infections. Potential risks related to local anesthesia do exist–including local anesthetic toxicity, allergic reactions, injection site pain or hematoma, and incomplete anesthesia. The use of epinephrine as a vasoconstrictor is typically well-tolerated, though caution may be exercised in those with severe vascular disease. Ultimately, WALANT avoids the systemic risks associated with general anesthesia and sedation, making it a low-risk and generally safe option for hand surgery [1,9].

Systematic reviews confirm no increased risk of infection or major adverse events outside the OR, given adherence to evidence-based sterility protocols.

## 9. Cost to Healthcare

Economic analyses report variable per case cost savings of USD 6–627 [14]. Alter et al. compared WALANT to sedated CTR and found a 45% reduction in cost when CTR is performed in a clinic setting rather than a traditional operating room [14]. A military medical center found an 85% reduction in facility costs (equivalent to approximately USD 627 savings per case), while outpatient clinics documented system-wide savings between USD 1320–1613 per case [9]. Nationally, shifting all 500,000 annual CTRs to clinic WALANT could potentially save over USD 750 million per year [13]. However, it is important to consider that the projected savings assume universal feasibility and do not consider implementation costs, patient suitability, cost variation, and regulatory constraints.

A time-driven activity-based cost (TDABC) analysis showed the average cost of in-office CTR was USD 152, compared with USD 557 in the ambulatory surgery center (ASC) [9]. This aligns with earlier military center data reporting 85% per case cost reductions [15]. Furthermore, clinic-based CTR procedures eliminate the need for pre-op clearance and postoperative recovery staffing, allowing a streamlined and cost-efficient workflow [15]. Ilyas et al. reported that transitioning to in-clinic WALANT enabled high-volume hand surgery throughput with reduced overhead and no compromise in outcomes [15].

Rhee et al. published a cost analysis of the first 100 consecutive clinic-based WALANT hand surgery procedures performed by a single surgeon at a military medical center. Performing procedures in the clinic under WALANT compared with the operating room resulted in a 70% cost savings [16].

The procedure is commonly billed under CPT code 64721, which applies regardless of surgical setting; however, reimbursement may vary depending on the place of service modifier and geographic region [4].

Economic advantages of the WALANT technique have been observed worldwide. A survey of the Argentinian Association of Hand Surgery compared the costs of CTR performed under WALANT techniques versus CTR performed under sedation in a conventional operating room. WALANT procedures reduced costs by over 64%. Despite these cost efficiencies, only 16% of surveyed Argentine hand surgeons preferred local anesthesia methods [17].

A retrospective review from the United Kingdom analyzed outcomes and costs of 18 patients undergoing WALANT CTR and found the techniques to be safe and cost-effective. In their study, patients experienced low pain (as reported on the visual analogue scale), minimal bleeding, and no wound complications. Most notably, authors reported a remarkably low overall cost per case of GBP 20, equivalent to approximately USD 21 [18].

In South Africa, the implementation of WALANT in outpatient settings resulted in significant financial benefits in a resource-strained public healthcare environment. Each WALANT procedure saved the institution between R18,892 and R27,962, equivalent to approximately USD 1220–1807 per case, compared to traditional operating room-based techniques. Additionally, WALANT reduced elective surgery waiting times from 3–4 months down to 6 weeks [19].

In a Spanish prospective study, costs were compared between 150 WALANT cases and 150 conventional surgeries. The authors found a substantial cost saving of GBP 1021 or USD 1073 per patient for the WALANT approach. Furthermore, WALANT increased patient throughput, allowing surgeons to perform eight cases per day versus five or six in the OR, freeing up 30 operating rooms, and cutting surgery wait times in half [20].

## 10. Clinical Implementation and Workflow

Efficient clinic-based CTR requires trained clinic staff, sterile procedure sets, and optimized turnover. In one study, procedure room turnover averaged 35 min faster per case versus OR, equating to 22–77 min saved per procedure when including elimination of anesthesia preparation and recovery time [3].

Moving CTRs to the clinic may liberate a significant portion of OR time previously occupied by simple hand surgeries. In practice, surgeons have reported increased capacity for complex cases in the OR following the adoption of clinic-based WALANT. By offloading CTS cases—which historically occupy a significant share of hand surgery block time—surgeons can optimize OR utilization across their practices.

## 11. Clinical Pearls and Additional Considerations

The use of virtual reality (VR) technology devices: A prospective trial of 59 WALANT hand surgery patients showed VR significantly reduced intra- and postoperative anxiety [21]. Another survey comparing office CTR vs. OR found VR further enhanced patient enjoyment and reduced anxiety in the office CTR group [5].

Offering noise-canceling headphones and music: In a randomized trial (N = 50), Kelly et al. showed that music and noise-canceling headphones decreased intraoperative anxiety (1.02 vs. 2.32 on a 10-point scale, *p* = 0.017) by 56%, with 92% of patients recommending the intervention to others [6].

Although technological devices can be an effective tool to reduce anxiety, it has several limitations. Patients may experience motion sickness, headaches, and certain medical conditions, such as seizure disorders, could preclude its use. Additionally, VR entails additional costs, the possibility of technical issues, and may require additional staff training.

## 12. Example Institutional Proposal

We propose a structured framework for surgeons and institutions seeking to implement WALANT CTR in the clinic setting. The following recommendations are derived from the published literature as well as practical insights from both civilian and military outpatient practices.

## 13. Institutional Proposal and Administrative Support

Successful implementation begins with administrative buy-in. Surgeons may consider submitting a formal request outlining the clinical rationale and economic advantages of in-clinic WALANT CTR. Such proposals should emphasize the procedure’s safety, high patient satisfaction, and demonstrated cost savings, often exceeding USD 600–1000 per case in facility fees alone. Highlighting the potential to decompress overburdened OR schedules may also strengthen institutional interest.

## 14. Room Setup and Instrumentation

The procedure is ideally performed in a standard outpatient procedure room under field sterility conditions. A minimal setup has been shown to be both safe and efficient. Required instruments typically include a scalpel, small retractor, scissors, Adson forceps, and a nerve hook. Skin preparation is performed with chlorhexidine, and a single sterile drape is used. Buffered lidocaine with epinephrine is injected subcutaneously into the carpal tunnel, with a 25–30 min delay for vasoconstriction. Additional supplies include sterile gloves, gauze, adhesive dressings, and optional comfort measures such as noise-canceling headphones or warming blankets.

## 15. Staff Training and Clinic Integration

Training clinical support staff is critical to ensuring smooth procedural flow and patient safety. Staff should be familiarized with:

- Field sterility principles;
- WALANT anesthetic administration and timing;
- Vasovagal response recognition and management;
- Documentation and consent workflows;
- Postoperative instructions and discharge protocols.

A brief in-service or shadowing of existing procedures is typically sufficient. Ensuring that staff understand the differences in workflow from traditional OR procedures will facilitate higher throughput and lower stress during the transition phase.

## 16. Scheduling and Workflow Efficiency

Once established, clinic-based WALANT CTR allows for high-volume, low-resource case throughput. A recommended schedule (Table 2) allocates approximately 60 min per case, including anesthesia setup, vasoconstriction delay, surgical time, and discharge. With this model, 3–4 cases can be completed in a half-day session. There is a potential for more cases as staff and surgeon familiarity improve. Efficient turnover is supported by simplified prep and the elimination of sedation-related recovery periods.

## 17. Billing and Compliance Considerations

WALANT CTR is typically billed under CPT code 64721, regardless of setting. Use of the “11” place of service modifier indicates clinic-based care. Institutions may require specific documentation templates capturing anesthesia details, sterility level, and postoperative instructions. Early engagement with the billing department is advisable to ensure appropriate reimbursement and avoid administrative barriers.


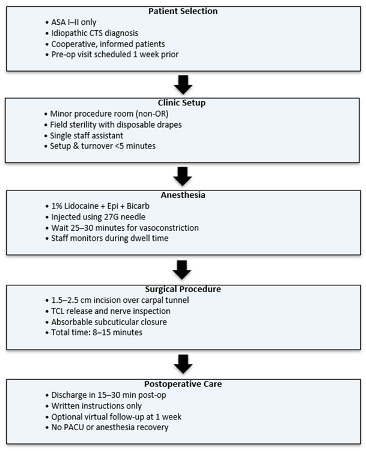


## 18. Future Directions

Wide-awake local anesthesia with no tourniquet (WALANT) carpal tunnel release in the clinic setting is well-supported by growing evidence, but large-scale implementation remains incomplete due to various barriers outlined below. To ensure sustainable adoption, future work should address not only cost and safety validation but also systems-level integration, education, and patient-centered innovation.

Although single-center studies demonstrate significant cost savings with WALANT CTR, comprehensive national economic modeling remains limited. Future research should evaluate cost implications across different payer structures, regions, and institutional sizes. This includes quantifying indirect cost savings from reduced need for anesthesia providers, recovery room staffing, preoperative testing, and OR time. Time-driven activity-based cost (TDABC) analyses, particularly those comparing various clinic types (e.g., academic vs. private practice), may provide actionable insights for administrators. Furthermore, integration with value-based payment models could incentivize WALANT adoption by aligning cost-efficiency with quality benchmarks.

Although the technique for carpal tunnel release offers advantages, not all patients are interested in this approach. In a randomized trial comparing bilateral carpal tunnel release under local-only anesthesia and sedation, 59% of patients preferred the local-only approach, while 34% preferred sedation, and 7% were indifferent. This reveals that approximately 41% of patients did not favor the WALANT technique [12]. In that case, it is important that surgeons continue to offer sedation or traditional anesthetic options alongside WALANT via shared decision-making and ensuring that treatment aligns with both patient comfort and clinical safety.

The standardization of credentialing and regulatory policies remains a major barrier. Many hospitals and surgery centers lack clear guidelines for office-based surgery, leading to hesitation among both administrators and surgeons. Future efforts should focus on establishing formal consensus recommendations for in-clinic WALANT procedures, including sterility protocols, safety checklists, and billing practices. These standards could be disseminated through professional societies such as ASSH or AAOS to guide institutional policy. Additionally, insurers and the Centers for Medicare and Medicaid Services (CMS) may consider revising reimbursement rules to reflect the unique structure and cost basis of in-clinic WALANT CTR, ensuring fair compensation for surgeons performing these high-value procedures outside the OR.

Surgical education must evolve to include WALANT-specific training. Despite its simplicity, successful clinic-based CTR requires mastery of field sterility, awake patient communication, and clinic workflow optimization. Residency and fellowship programs should incorporate WALANT modules to ensure that trainees are proficient not only in the surgical technique but also in patient selection, documentation, and post-op management without anesthesiology support. Simulation-based models or cadaver labs could offer safe, structured environments for learning these skills.

Summary of Current Barriers to Widespread Implementation

- Lack of comprehensive national economic modeling;
- Absence of consensus recommendations from professional societies;
- Need for standardized credentialing and regulatory policies across healthcare systems;
- Revisions to insurance reimbursement to ensure fair compensation;
- Expansion of WALANT CTR within surgical education to ensure proficiency.

As WALANT becomes more mainstream, long-term outcome data will be essential to support its durability and safety. While early studies confirm low complication rates and high patient satisfaction, future prospective registries and multicenter cohort studies should track infection rates, functional recovery, and recurrence over years—not weeks. Comparative studies assessing outcomes between office-based and OR-based CTR for specific subpopulations (e.g., diabetic patients, revision cases, or those with high BMI) would help refine patient selection criteria.

As the clinic-based WALANT CTR model matures, opportunities exist to improve the patient experience. The use of immersive technologies such as virtual reality, noise-canceling headsets, and digital pain tracking apps should be formally studied in the context of awake surgery. Similarly, assessing patient expectations, cultural concerns, and communication preferences can help develop a more holistic, patient-centered approach to awake procedures.

## 19. Conclusions

WALANT carpal tunnel release performed in the clinic is a safe, cost-effective, and patient-friendly procedure with comparable outcomes to traditional OR-based surgery. Strategic patient selection, efficient clinical workflows, and adjunctive comfort measures allow surgeons to improve OR utilization, reduce healthcare costs, and enhance patient satisfaction. Implementation in practice has many hurdles, but the literature continues to build and support clinic-based hand surgery. This has the potential to improve the patient experience, surgeon efficiency, and the overall cost to healthcare. Adoption of clinic-based WALANT CTR has the potential to transform hand surgery delivery across multiple practice settings worldwide.

## Figures and Tables

**Table 1:** Patient selection criteria for clinic-based WALANT CTR.

Inclusion	Exclusion
- ASA I–II - Idiopathic carpal tunnel syndrome confirmed clinically or with electrodiagnostic studies - Cooperative patient comfortable with awake surgery - No prior complex hand or wrist surgery	- ASA III+ or significant cardiopulmonary disease - Revision CTR or abnormal wrist anatomy - Active psychiatric or anxiety disorders precluding cooperation - Language or cognitive barriers preventing informed consent

ASA: American Society of Anesthesiology score. CTR: carpal tunnel release.

**Table 2:** Time estimates for each phase of the WALANT CTR protocol.

Phase	Estimated Time
Patient check-in	10 min
Local anesthetic setup	5 min
Vasoconstriction delay	25–30 min
Procedure time	10–15 min
Discharge and turnover	10–15 min
